# 516. Development of a Cold Induced Burn Injury Model in Swine

**DOI:** 10.1093/jbcr/irag033.175

**Published:** 2026-04-06

**Authors:** Adam J Singer, Rachel B Bonn, Shi Fu, Huiting Luo, Zachery B Harris, Erica Heller, Steve McClain, Robert Ruzic, Briman Yang, Miriam Rafailovich, Gurtej Singh, Marcia Simon, Steven Sandoval, Ulas Sunar, Arash Karimi, M Hassan Arbab, Elias Kluiszo, Irfaan A Dar, Lior Rosenberg

**Affiliations:** Stony Brook University, Smithtown, NY; SUNY Stony Brook, Stony Brook, NY; SUNY Stony Brook, Stony Brook, NY; SUNY Stony Brook, Stony Brook, NY; SUNY Stony Brook, Stony Brook, NY; SUNY Stony Brook, Stony Brook, NY; SUNY Stony Brook, Stony Brook, NY; SUNY Stony Brook, Stony Brook, NY; SUNY Stony Brook, Stony Brook, NY; SUNY Stony Brook, Stony Brook, NY; SUNY Stony Brook, Stony Brook, NY; SUNY Stony Brook, Stony Brook, NY; SUNY Stony Brook, Stony Brook, NY; SUNY Stony Brook, Stony Brook, NY; SUNY Stony Brook, Stony Brook, NY; SUNY Stony Brook, Stony Brook, NY; SUNY Stony Brook, Stony Brook, NY; SUNY Stony Brook, Stony Brook, NY; Ben Gurion University, Beer Sheva, HaDarom

## Abstract

**Introduction:**

Cold-induced burns include a range of injuries from subfreezing frostbite to non-freezing cold injuries. Development of novel therapies has been hindered by poor understanding of the mechanisms leading to injury progression. We developed a porcine experimental model of cold-induced injuries and explored the underlying mechanisms contributing to injury progression.

**Methods:**

Cold injuries were created on the dorsal and ventral surfaces of 4 anesthetized pigs using regular ice (0°C), dry ice (-78°C) and liquid nitrogen (LN, -198°C). Forward Looking Infrared (FLIR) imaging measured post-injury wound temperature. Blood flow index (BFI) was measured with Laser Speckle Contrast Imaging (LSCI) and Diffuse Correlation Spectroscopy (DCS), and wound biopsies were collected to monitor depth of injury and scarring over 28 days.

**Results:**

Exposure to regular ice (30-60 min) caused temporary erythema that completely resolved within 1 hour with no subsequent injury. Dry ice exposure (1-5 min) led to temporary erythema and superficial dermal injury that completely healed within several days. Exposure of skin to LN for 30-60 sec resulted in temporary superficial freezing with a reduction in skin temperature to below -40°C over the wound center. These areas were characterized by cellular necrosis of endothelial cells and dermal appendage epithelial cells. More peripheral areas subject to near freezing temperatures (0-4°C) were characterized by plugging of blood vessels with red blood cells (RBC) and fibrin throughout the dermis and subcutaneous fat. Percentage of fully reepithelialized wounds at 21 days for 30 and 60 second exposures were 75% vs. 0% (*p*=.03). The 30-second injury displayed an initial increase in BFI at 30 minutes followed by a decrease at 120 minutes, while the 60-second injury showed a consistent decline in BFI over time. In vitro exposure of fibrinogen to near freezing temperatures resulted in cryoprecipitation of fibrin that was reversed with a fibronectin derived peptide (P12) that reduces thermal burn injury progression.

**Conclusions:**

Cold induced injuries are consistently produced by exposing the pigs to LN for 30-60 seconds. Significant cellular necrosis was noted especially in the center of the wounds exposed to subzero temperatures. More peripheral areas exposed to non-freezing cold temperatures were characterized by a reduction in cutaneous blood flow due to plugging of blood vessels with RBC and precipitated fibrin. In vitro data suggest P12 may reduce fibrin precipitation at near zero temperatures.

**Applicability of Research to Practice:**

Our results suggest that cold induced injuries are secondary to cellular necrosis as well as blood vessel plugging with RBC and fibrin, which can be partially reduced, in vitro, with P12. Future studies will determine whether treatment with P12 can reduce cold injury progression in the porcine model.

**Funding for the study:**

Supported by the Suffolk County Volunteer Firefighters Burn Fund.

